# Pharmacological profiling of intravenous MP-04: sustained NAD^+^ augmentation, immune modulation, and renal protection in preclinical models

**DOI:** 10.3389/fphar.2026.1832979

**Published:** 2026-06-10

**Authors:** Alip Ghosh, Gautham Pranesh, Nikhil Zachariah, Eisuke Murakami, Alonso Heredia, Poonam Mathur, Leigh Ann Burns-Naas, Steve Weng, Arvind Ramanathan, Shyam Kottilil, G. Mani Subramanian

**Affiliations:** 1 Institute of Human Virology, University of Maryland School of Medicine, Baltimore, MD, United States; 2 MitoPower, LLC, Palo Alto, CA, United States; 3 Magnolia Toxicology Consulting, Limited Liability Company, Michigan, MI, United States; 4 Institute for Stem Cell Science and Regenerative Medicine (InStem), Bangalore, India

**Keywords:** acute kidney injury (AKI), cytokine modulation, dihydronicotinamide riboside (NRH), hepatorenal syndrome, immunomodulation, mitochondrial bioenergetics, NAD+ metabolism, systemic inflammation

## Abstract

**Introduction:**

Systemic inflammation and mitochondrial bioenergetic failure are central drivers of multi-organ dysfunction in advanced liver disease, sepsis, and acute kidney injury (AKI). Currently available therapies remain largely supportive and fail to directly address intracellular NAD^+^ depletion and immune-metabolic dysregulation. We investigated MP-04, a novel intravenous formulation of dihydronicotinamide riboside (NRH), for its ability to restore NAD^+^ homeostasis, modulate immune metabolism, and confer organ protection in relevant preclinical models.

**Methods:**

The NAD^+^-enhancement activity of nicotinamide riboside (NR) and MP-04 was evaluated in human hepatoma (HepG2), T-cell (Jurkat), kidney (HEK293) cell lines, and peripheral blood mononuclear cells (PBMCs). Cellular bioenergetics were assessed using Seahorse extracellular flux analysis. Immunomodulatory effects were examined in polyclonally activated human PBMCs and in a murine endotoxin-induced systemic inflammation model. Pharmacokinetics and pharmacodynamics were assessed following intravenous (IV) dosing in rats, and organ protection was evaluated in a cisplatin-induced AKI mouse model. Safety of MP-04 was evaluated in rat and dog Good Laboratory Practice (GLP) toxicology studies.

**Results:**

MP-04 produced rapid, distinct, and dose-dependent increases in intracellular NAD^+^ across all tested human cell types and was markedly more potent than NR. IV administration in rats resulted in sustained elevations of NAD^+^ and NADH in blood, liver, and kidney that persisted beyond the systemic clearance of MP-04, with strong correlations between blood and tissue NAD^+^ levels. In activated immune cells, MP-04 reduced reliance on aerobic glycolysis, and significantly attenuated pro-inflammatory cytokine production without affecting resting cells. *In vivo*, MP-04 reduced systemic inflammatory cytokines following endotoxin challenge and conferred significant biochemical and histological protection against cisplatin-induced AKI. MP-04 was safe and well tolerated in rat and dog following once daily IV administration for 7 days.

**Conclusion:**

MP-04 is a safe and highly potent intravenous NAD^+^ precursor that modulates cellular metabolism, reduces maladaptive immune activation, and protects against inflammation-associated organ injury in preclinical models. These findings support MP-04 as a promising metabolic-immunomodulatory therapeutic strategy for conditions characterized by systemic inflammation and organ failure, including hepatorenal syndrome–associated AKI, beyond the limits of current supportive care.

## 1 Introduction

Nicotinamide adenine dinucleotide (NAD^+^) is a crucial coenzyme that plays vital roles in cellular metabolism, energy production, and the maintenance of genomic integrity (Canto et al., 2015; Verdin, 2015; Covarrubias et al., 2021). Clinical and preclinical studies have increasingly revealed that low NAD^+^ levels are associated with various forms of organ dysfunction and systemic inflammation (Verdin, 2015; Covarrubias et al., 2021; Yusri et al., 2025). Particularly in conditions such as acute kidney injury (AKI) and sepsis, where an exaggerated inflammatory response contributes to organ failure, restoring NAD^+^ levels emerges as an important therapeutic strategy (Brealey et al., 2002; Hotchkiss et al., 2016; Alhumaidi et al., 2025). By promoting cellular repair mechanisms and modulating inflammatory pathways, NAD^+^ precursors can potentially reduce tissue damage and improve organ function (Pearce et al., 2013; O'Neill et al., 2016; Tran et al., 2016; Buck et al., 2017; Poyan Mehr et al., 2018).

Among the emerging NAD^+^ precursors, nicotinamide riboside (NR) has been widely studied for their ability to augment intracellular NAD^+^ levels (Canto et al., 2012; Yoshino et al., 2018); however, recent evidence suggests that dihydronicotinamide riboside (NRH) presents several advantages that may enhance its therapeutic efficacy (Biggins et al., 2021; Zhang et al., 2025). Unlike NR, NRH is uncharged, facilitating superior cellular absorption and transport across biological membranes. Additionally, NRH is metabolically converted to NAD^+^ more efficiently through the activity of adenosine kinase, leading to significantly higher NAD^+^ concentrations compared to NR (Yang and Sauve, 2016; Giroud-Gerbetant et al., 2019). These properties collectively position NRH as a promising candidate for more impactful modulation of cellular functions and metabolic pathways linked to stress responses and inflammation.

In this report, we investigated the characteristics and therapeutic potential of MP-04, an innovative intravenous formulation of NRH. Our research focuses on evaluating MP-04’s ability to elevate NAD^+^ levels and modulate immune responses *in vitro*, as well as its translational effectiveness in preclinical rodent models of AKI and LPS-induced sepsis. Here our aim was to elucidate the immune modulatory effects of MP-04 and explore its implications for enhancing NAD^+^ bioavailability and its protective roles during acute inflammatory episodes. Through this comprehensive analysis, we looked to establish a foundation for the application of NRH in critical care and to address the pressing need for effective therapeutic strategies to mitigate organ dysfunction and inflammation.

## 2 Materials and methods

### 2.1 MP-04 formulation

MP-04 drug substance (DS) was synthesized in a 3-step proprietary process with an overall yield of 58%. MP-04 DS was then formulated into an intravenous formulation of dihydronicotinamide riboside developed by MitoPower, LLC, and designed to enable rapid and predictable systemic NAD^+^ augmentation. The formulation exhibits acceptable aqueous stability to preserve the chemical stability during storage and intravenous administration, supporting reproducible pharmacokinetic and pharmacodynamic evaluation in preclinical models.

### 2.2 Intracellular NAD^+^ quantification in cell lines

Intracellular NAD^+^ levels were quantified in HepG2, Jurkat, and HEK293 cells following treatment with MP-04 and NR. HepG2 human hepatocellular carcinoma cells were cultured in DMEM supplemented with 10% fetal bovine serum and 1% penicillin–streptomycin and seeded at 0.5 × 10^6^ cells per well in a 96-well, clear-flat bottom tissue culture plate. Jurkat T lymphocyte cells were maintained in RPMI-1640 supplemented with 10% fetal bovine serum and 1% penicillin–streptomycin and seeded at 1.0 × 10^6^ cells per well in 48 well clear flat bottom, tissue culture, sterile plates. HEK293 human embryonic kidney cells were cultured in serum-containing DMEM and seeded at densities sufficient to achieve ∼80% confluence at the time of treatment. All cell lines were incubated at 37 °C in a humidified atmosphere containing 5% CO_2_ and allowed to stabilize for 18–24 h prior to dosing. All these cells were incubated with 40 µM Methylnitronitrosoguanidine (MNNG) for 30 min to induce cell damage. Cells were treated with MP-04 or NR over concentration ranges of 5–250 µM (HepG2), 5–1000 µM (Jurkat), and 0.01–1000 µM (HEK293). HepG2 and Jurkat cells were exposed to the test compounds (MP-04 or NR) for 4 h, while HEK293 cells were treated for 6 h under serum conditions.

NAD^+^ levels were measured in HepG2 & Jurkat cells using a liquid chromatography–mass spectrometry (LC-MS)/MS based assay. Following treatment, HepG2 and Jurkat cells were rapidly washed with ice-cold phosphate-buffered saline and extracted using cold methanol-based solvent systems optimized for preservation of nucleotides. Clarified extracts were analyzed by LC-MS using a Thermo Vanquish UHPLC system coupled to a high-resolution Orbitrap mass spectrometer (Thermo Q-Exactive Orbitrap) operating primarily in positive electrospray ionization mode. NAD^+^ and NADH levels were quantified from integrated peak areas relative to internal standards (Tolbutamide) and calculated the absolute concentrations using calibration curves generated with reference standards. Each condition was analyzed in technical triplicate, and replicate means were used for downstream analysis. In HEK293 cells, total intracellular NAD (NAD^+^ + NADH) was quantified using a luciferase-coupled enzymatic cycling luminescence assay. Following compound exposure, cells were lysed directly in assay buffer, and luminescent signal proportional to intracellular NAD content was measured using a multimode plate reader. Background signal from media-only wells was subtracted, and results were expressed relative to untreated control cells. Across all assays, data quality was assessed by replicate variability (coefficient of variation), and no outliers were excluded unless analytically justified. These methodologies enabled consistent, quantitative comparison of NAD-elevating activity of MP-04 and NR across multiple cell types and analytical platforms.

### 2.3 Human samples and PBMC isolation

Leukocyte samples from healthy donors were obtained from the New York Blood Center. Peripheral blood mononuclear cells (PBMCs) were isolated by Ficoll-Paque density gradient centrifugation (Sigma-Aldrich), resuspended in 90% fetal bovine serum (Gibco) with 10% DMSO, and cryopreserved in liquid nitrogen vapor phase until use.

### 2.4 Intracellular NAD^+^ quantification in PBMCs

PBMCs (1 × 10^6^ cells/well, in duplicate) from five different donors were treated with MP-04 or NR (250 μM) for 3 h. NAD^+^ levels were measured using the NAD/NADH-Glo™ Assay (Promega) according to the manufacturer’s protocol and normalized to the cell number.

### 2.5 Cell viability

Cell viability following MP-04 treatment (250 μM for 4 h) in PBMCs from two healthy donors was assessed by flow cytometry using Zombie-NIR viability dye (BioLegend).

### 2.6 Pharmacokinetics-pharmacodynamics (PK-PD) of MP-04 in rat and dog

The PK-PD of MP-04 were evaluated following intravenous administration in rats and dogs. All studies were conducted in compliance with applicable institutional and ethical guidelines for animal research. Male Sprague–Dawley rats were dosed with MP-04 via intravenous bolus administration at doses of 50, 125, or 250 mg/kg. Blood samples were collected at pre-dose, 0.5, 1, 2, 4, 8, 12, and 24 h post-dose for determination of whole-blood MP-04 concentrations in K2EDTA tubes via the jugular vein catheter. In a subset of animals, a second intravenous dose was administered 24 h after the initial dose, and whole blood, liver, and kidney samples were collected 4 h post-dose to assess tissue exposure and pharmacodynamic effects on NAD^+^ and NADH levels. MP-04, NAD^+^, and NADH concentrations in whole blood and tissue samples were quantified using a qualified LC–MS/MS method. Samples were stored at −80 °C, thawed at 2 °C–8 °C, and processed under cold conditions to preserve analyte stability. Whole blood samples were subjected to protein precipitation with ice-cold methanol containing rac-tenofovir-D6 as an internal standard, followed by centrifugation (3000 g, 15 min, 4 °C) and analysis of the clarified supernatant. Tissue samples (liver and kidney) were homogenized in a 1:1 water:methanol solution (1 g:19 mL) and centrifuged to obtain extracts. Calibration standards and quality controls were prepared in 5% bovine serum albumin as a surrogate matrix. Chromatographic separation was performed on a C18 column (Waters XBridge) at 40 °C using a gradient of ammonium acetate–based mobile phases at a flow rate of 0.5 mL/min (6 min run time). Detection was carried out on a triple quadrupole mass spectrometer (SCIEX Triple Quad™ 4,500) with positive electrospray ionization in multiple reaction monitoring mode. Pharmacokinetic parameters (Cmax, Tmax, AUC, and T1/2) were derived by non-compartmental analysis using Phoenix WinNonlin (Certara United States of America, Inc.).

The PK-PD study in male and female dogs was conducted following IV infusion of MP-04 at 0 (vehicle), 75, 100, and 300 mg/kg (n = 3 or 4). In-life phase of this study was conducted at Inotiv (Mt. Vernon, IN, United States of America) and bioanalysis was performed at Pyxant Labs, Inc. (Colorado Springs, CO, United States of America). Blood samples were collected at pre-dose, 0.083, 0.25, 0.33, 0.5, 1, 2, 4, 8, and 24 h post-dose relative to beginning of infusion in K2EDTA tube via peripheral vessel.

The MP-04 concentrations in biological matrices were quantified using qualified liquid chromatography–tandem mass spectrometry (LC–MS/MS) methods. Whole-blood and tissue NAD^+^ and NADH levels were quantified using a qualified LC-MS/MS bioanalytical method. For the standard curves, 5% BSA was used as a surrogate matrix. Pharmacokinetic parameters, including maximum observed concentration (Cmax), time to maximum concentration (Tmax), area under the concentration–time curve (AUC), and terminal half-life (T1/2), were calculated by non-compartmental analysis using Phoenix WinNonlin software (Certara United States of America, Inc.).

### 2.7 7-Day repeat dose good laboratory practice (GLP) toxicity studies

The toxicity of MP-04 following daily intravenous administration for 7 days was evaluated in Wistar Han (WH) rats and Beagle dogs in compliance with the current U.S. FDA GLP Regulations for Non-clinical Studies. For both rats and dogs, MP-04 was formulated in 20 mM Na_2_HPO_4_ and 20 mM NaCl (pH 9.5) and vehicle was formulated in 20 mM Na_2_HPO_4_ and 120 mM NaCl.

#### 2.7.1 Rat toxicity study

These studies were conducted at Inotiv (Gaithersburg, MD, United States of America) with approval from IACUC at Inotive. MP-04 was administered daily via IV injection for 7 days at dose levels of 0 (vehicle), 50, 100, and 200 mg/kg to Wistar Han rats (5/sex/group for vehicle group and 10/sex/group for treatment groups) to establish the systemic toxicity profile. Animals were evaluated for cage side observation, physical examinations, body weights, food consumption, clinical pathology (hematology, coagulation, chemistry, and urinalysis), ophthalmologic examination, functional observational battery (FOB), and pulmonary measurements. Toxicokinetic (TK) analysis was conducted in a separate group (3/sex/group for vehicle group and 6/sex/group for treatment groups) on Day 1 and Day 7. TK samples were analyzed for MP-04 using a validated method in rat plasma. On Day 8, animals were sacrificed for macroscopic pathology, bone marrow slides, organ weights, and histology and microscopic pathology.

#### 2.7.2 Dog toxicity study

These studies were conducted at Inotiv (Mt. Vernon, IN, United States of America) with approval from IACUC at Inotive. MP-04 was administered daily via IV injection for 7 days at dose levels of 0 (vehicle), 10, 30, and 60 mg/kg to dogs (4/sex/group) to establish the systemic toxicity profile. Animals were evaluated for clinical observation, physical examinations, body weights, food consumption, clinical pathology (hematology, coagulation, chemistry, and urinalysis), ophthalmologic examination, and electrocardiograms. Toxicokinetic (TK) analysis was conducted on Day 1 and Day 7. TK samples were analyzed for MP-04 using a validated method in dog plasma. On Day 8, animals were sacrificed for macroscopic pathology, bone marrow slides, organ weights, and histology and microscopic pathology.

### 2.8 Cellular metabolic activity

Mitochondrial respiration and glycolysis were measured using the Seahorse XF extracellular flux analyzer (Agilent) and Mito Stress Test Kit (Agilent). PBMCs were stimulated with anti-CD3 (1 μg/mL) and anti-CD28 (5 μg/mL) antibodies with or without MP-04 (250 μM) for 24 h. Cells were seeded onto Cell-Tak–coated plates at 5 × 10^5^ cells/well and incubated in XF RPMI medium supplemented with glucose (10 mM), glutamine (2 mM), and pyruvate (1 mM). Basal oxygen consumption rate (OCR) and extracellular acidification rate (ECAR) were measured, followed by sequential addition of oligomycin (1 μM), FCCP (1.5 μM), and rotenone/antimycin A (0.5 μM). ATP-linked respiration and spare respiratory capacity were calculated according to manufacturer’s protocols.

### 2.9 Intracellular cytokine measurement

Cryopreserved human PBMCs were thawed and incubated for 16–18 h in complete RPMI 1640 medium. Cells were stimulated with immobilized anti-CD3 (1 μg/mL) and soluble anti-CD28 (5 μg/mL) antibodies (ThermoFisher Scientific) in the presence or absence of NR or MP-04 (250 μM) for 24 h. Unstimulated cells served as controls. Cells were stained with antibodies against CD3 (FITC), CD4 (BV421), and CD8 (BV510) (BioLegend), permeabilized using Cytofix/Cytoperm (BD Biosciences), and stained intracellularly for IFNγ (APC), TNFα (PE), and IL-2 (PerCP/Cy5.5) (BioLegend). Live/dead discrimination was performed using Zombie-NIR dye. Data were acquired on an LSR-II flow cytometer (BD Biosciences) and analyzed using FlowJo v10.7.1.

### 2.10 Mouse model of acute kidney injury

Effect of MP-04 in mouse acute kidney injury model was evaluated at TheraIndx (Bengaluru, India) with approval from IAEC at TheraIndx. Female BALB/c mice (six to eight weeks) received a single intraperitoneal injection of 25 mg/kg cisplatin (Cat. #D3371, TCI Chemicals) to induce acute kidney injury. MP-04 (150 mg/kg) or vehicle (PBS) was administered intraperitoneally once daily for 6 days (3 days before and 3 days after cisplatin). Body weight was monitored throughout the study. Animals were sacrificed 12 h after the final dose. Serum creatinine and blood urea nitrogen (BUN) were measured, and kidneys were collected from each animal, fixed in 10% NBF (Neutral Buffered Saline) or formalin and subjected to histopathology. Hematoxylin and Eosin (H&E), and periodic acid–Schiff (PAS) staining of 4 micron-thick tissue sections were performed according to the standard protocols.

### 2.11 Mouse model of endotoxin-induced inflammation

Lipopolysaccharide (LPS) was used to induce inflammation in male C57BL/6 mice. Mice were randomized into four groups (n = 3/group): PBS/PBS, MP-04/PBS, PBS/LPS, and MP-04/LPS. MP-04 (250 mg/kg) or PBS was administered intraperitoneally, followed by LPS (5 mg/kg) or PBS after 1 h. Blood was collected 6 h later, and plasma cytokines and chemokines (IFNγ, IL-1β, RANTES, IP-10, MCP-1, IL-6, IL-10, IL-12) were quantified using multiplex assays (Luminex). The mice study was approved by IACUC, University of Maryland School of Medicine, Baltimore.

### 2.12 Statistical analysis

Statistical analyses were performed using GraphPad Prism version 10.0.2 for Windows (GraphPad Software, San Diego, CA, United States of America). Data are presented as mean ± SEM unless otherwise indicated. Each dot represents an individual biological replicate (donor or animal), as specified in the figure legends. Comparisons between two groups were performed using paired or unpaired two-tailed Student’s T-tests, as appropriate. For experiments involving more than two groups, one-way ANOVA followed by multiple-comparison *post hoc* testing was used. Correlations between blood and tissue NAD^+^ levels were assessed using linear regression analysis, and goodness-of-fit is reported as *R* ^2^. A p-value <0.05 was considered statistically significant. Statistical significance in figures is indicated as follows: *p < 0.05, **p < 0.01, ***p < 0.001; ns, not significant.

## 3 Results

### 3.1 MP-04 increases NAD levels in different cell types *in vitro*

**FIGURE 1:**
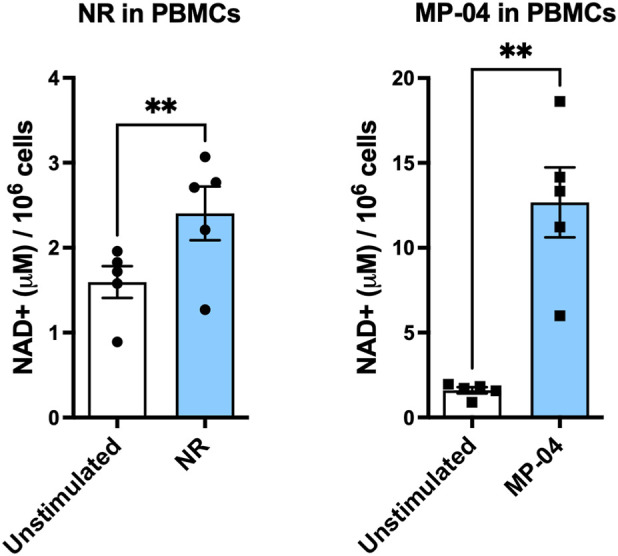
MP-04 markedly increases intracellular NAD^+^ levels in human PBMCs. Peripheral blood mononuclear cells (PBMCs) from five healthy donors were treated with MP-04 (250 μM) or nicotinamide riboside (NR; 250 μM) for 4 h. Intracellular NAD + levels were quantified and normalized per million cells. Each dot represents one donor. Statistical significance was determined using paired t-test. **p < 0.01; ***p < 0.001. US, unstimulated.

**FIGURE 2:**
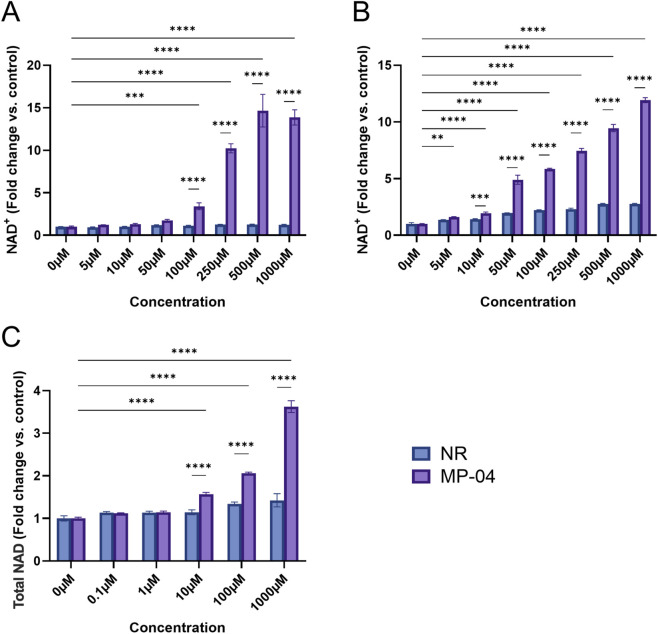
MP-04 increases intracellular NAD^+^ in a dose-dependent manner across human cell lines. **(A)** HepG2 cells treated with MP-04 (5–250 μM) for 4 h demonstrate dose-dependent increases in NAD^+^ levels. **(B)** Jurkat T cells treated with MP-04 (5–1000 μM) for 4 h show dose-dependent intracellular NAD^+^ elevation. **(C)** HEK293 cells treated with MP-04 (0.1–1000 μM) exhibit increased total NAD (NAD^+^ + NADH). NAD^+^ levels in HepG2 and Jurkat cells were quantified by LC–MS/MS; total NAD in HEK293 cells was measured using a luminescence-based assay. Data are presented relative to untreated controls. Statistical analysis was performed using one-way ANOVA with multiple comparison correction. **p < 0.01, ***p < 0.001, ****p < 0.0001 versus untreated.

Intracellular levels of NAD^+^ were determined following incubation of human PBMCs from five different donors with 250 μM MP-04 or NR for 4 h. As shown in Figure 1, NR treatment resulted in an increase in intracellular NAD^+^ levels at 4 h compared with unstimulated controls (approximately 1.5-fold increase; p < 0.01). In contrast, MP-04 produced a markedly greater increase of intracellular NAD^+^ levels at 4 h (Figure 1). Treatment increased NAD^+^ concentrations from a median of 1.72 μM (range 0.89–1.96) in unstimulated controls to 13.34 μM (range 6.00–18.63); representing an approximately 7–8-fold increase (**p < 0.01**). Cell viability was maintained with no detectable cytotoxicity observed at concentrations up to 1 mM (Supplementary Figure S1). Intracellular levels of NAD^+^ were further evaluated in hepatic cell line (HepG2 cells), T lymphocyte cell line (Jurkat cells), and total intracellular NAD (NAD^+^ + NADH) levels were evaluated in kidney derived HEK293 cells after treatment with NR or MP-04. Increases in NAD^+^ and total NAD levels were observed with NR incubation with up to 1.3-, 2.8-, and 1.4-fold in HepG2, Jurkat, and HEK293 cells, respectively. In contrast, significant and dose-dependent increases in intracellular NAD^+^ and total NAD levels were observed with up to 15-, 12-, and 3.6-fold in HepG2, Jurkat, and HEK293 cells, respectively (Figure 2). In addition, MP-04 exerts cytoprotective effects under conditions of NAD^+^ depletion and DNA damage (Supplementary Figure S5). MP-04 was tested in Jurkat and HepG2 cells exposed to the DNA-alkylating agent MNNG. In Jurkat cells, MNNG induced marked cytotoxicity, whereas MP-04 restored cell viability to near-baseline levels in a dose-dependent manner (EC_50_ = 40.3 µM). In HepG2 cells, exposure to 600 µM MNNG followed by treatment with MP-04 (10–1000 µM) resulted in robust, dose-dependent cytoprotection as measured by Alamar Blue/Cell Titer-Blue assays. Notably, MP-04 was significantly more potent than nicotinamide riboside (NR) (p < 0.05) in mitigating MNNG-induced cytotoxicity (Supplementary Figure S2).

### 3.2 MP-04 increases NAD^+^ levels in rats and dogs *in vivo*

Pharmacokinetics and pharmacodynamic effects of MP-04 were evaluated in rats and dogs following intravenous administration of MP-04. In rats, pharmacokinetics of MP-04 was determined following a single intravenous administration at 50, 125, and 250 mg/kg. MP-04 exhibited rapid systemic clearance, with a short terminal half-life ranging from 0.2 to 0.3 h, and was below the limit of quantification in whole blood by 2 h post-dose (Supplementary Figure S3; Table 1). To assess pharmacodynamic effects, NAD^+^ and NADH levels were measured in whole blood, liver, and kidney following repeat dosing (0 and 24 h), with tissue collection performed 4 h after the second dose. Dose-dependent increases in NAD^+^ and NADH levels were observed across all compartments (Supplementary Figure S4). The NAD+/NADH ratios were also evaluated in these tissues and the ratio increased dose-dependently in whole blood but no significant changes were observed in liver and kidney (Supplementary Figure S4). Notably, these increases were detected at time points when circulating MP-04 concentrations were minimal or undetectable (Supplementary Figure S3). A strong correlation was observed between whole-blood NAD^+^ levels and tissue NAD^+^ concentrations in liver (*R* ^2^ = 0.8592, p = 0.0001) and kidney (*R* ^2^ = 0.7874, p = 0.0003), indicating that circulating NAD^+^ levels are associated with tissue NAD^+^ levels under these experimental conditions (Supplementary Figure S5).

**TABLE 1:** PK parameters of MP-04 following a single IV administration to rats and dogs.

Species	Rat^*^			Dog		
Dose (mg/kg)	50	125	250	75	100	300
T_max_ (h)	0.50 ± 0.00	0.50 ± 0.00	0.50 ± 0.00	0.187 ± 0.125	0.172 ± 0.126	0.245 ± 0.025
C_max_ (ng/mL)	18.2 ± 9.2	74.1 ± 12.0	282 ± 26	10,200 ± 3,600	23,300 ± 8,900	85,800 ± 19,200
AUC_0-t_ (h*ng/mL)	6.12 ± 2.38	39.5 ± 6.2	169 ± 11	3,620 ± 650	5,940 ± 2,220	30,900 ± 2,600
T_1/2_ (h)	NC	0.173 ± 0.012	0.328 ± 0.033	0.157 ± 0.046	0.205 ± 0.026	0.439 ± 0.041

^*^ The lower exposures to MP-04 in rat are an underestimation and due to delayed first PK sample collection (0.50 h postdose) post IV administration.

In dogs, following single intravenous administration at doses of 75, 100, and 300 mg/kg, MP-04 similarly demonstrated rapid clearance from whole blood, with terminal half-lives ranging from 0.16 to 0.44 h (Supplementary Figure S6; Table 1). Concurrently, NAD^+^ levels increased within minutes of administration relative to baseline (predose) and remained elevated throughout the 24-h observation period (Supplementary Figure S6).

### 3.3 Safety of MP-04 in rats and dogs

To support the clinical development of MP-04, safety of MP-04 was evaluated in rats and dogs following daily intravenous administration for 7 days under GLP compliance. In a 7-day repeat-dose toxicity study in female and male rats, MP-04 was administered via IV bolus once daily at 0 (vehicle), 50, 100, and 200 mg/kg/day. Treatment was generally well tolerated up to 200 mg/kg/day. Clinical pathology and macroscopic and microscopic evaluations at the 200 mg/kg/day dose revealed no adverse effects on hematology, clinical chemistry (including liver enzymes), coagulation, or histopathology in any organ examined. Dedicated assessments for safety pharmacology components—CNS/behavioral, and respiratory function—similarly showed no toxicologically significant effects related to MP-04 administration across all dose groups (Data not shown). Based on the absence of mortality, significant adverse clinical signs, or pathological findings, the No-Observed-Adverse-Effect Level (NOAEL) was established at the highest dose tested: 200 mg/kg/day for both female and male WH rats. Systemic exposure (Cmax and AUC_last_) to MP-04 at this NOAEL was high, reaching 140,000 ng/mL and 41,500 ng*h/mL, respectively, in females and 165,000 ng/mL and 54,000 ng*h/mL, respectively, in males (Data not shown). The human equivalent dose at the NOAEL in rat (200 mg/kg) is 2,260 mg which is 4.5-fold higher than the highest intended dose of 500 mg in Phase 1 studies.

In a 7-day repeat-dose toxicity study in female and male dogs (4/sex/group), MP-04 was administered once daily via IV infusion at a rate of 2 mL/min for seven consecutive days, at dosages of 0 (vehicle), 10, 30, and 60 mg/kg/day. The study demonstrated that MP-04 was well tolerated across all dose groups, resulting in no treatment-related changes in any parameter measured, including detailed clinical observations, body weights, food consumption, ophthalmology findings, electrocardiogram parameters, or clinical pathology (hematology, coagulation, clinical chemistry, and urinalysis). Furthermore, post-mortem examinations, including macroscopic pathology, absolute and relative organ weights, bone marrow smears, and comprehensive microscopic pathology, revealed no MP-04-related findings at any dose level. Therefore, the NOAEL for the 7-day repeat-dose IV administration was established at the highest dose tested, 60 mg/kg, for both female and male Beagle dogs. Systemic exposure (C_max_ and AUC_last_) to MP-04 at this NOAEL was high, reaching 111,000 ng/mL and 31,500 ng*h/mL, respectively, in females and 87,100 ng/mL and 24,500 ng*h/mL, respectively, in males (Data not shown). The human equivalent dose at the NOAEL in dog (60 mg/kg) is 2,300 mg which is 4.7-fold higher than the highest intended dose of 500 mg in Phase 1 studies.

### 3.4 MP-04 alters extracellular acidification responses in activated T cells

**FIGURE 3:**
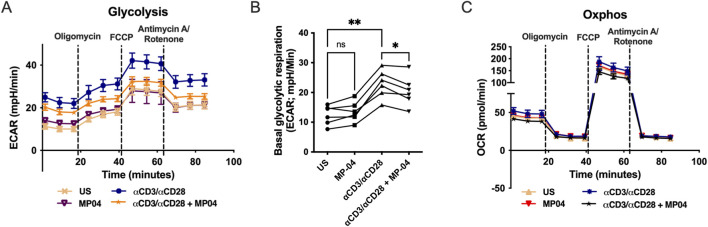
MP-04 modulates metabolic responses in activated human T cells. **(A)** Real-time extracellular acidification rate (ECAR) measured using the Seahorse XF Mito Stress Test in unstimulated (US) or anti-CD3/anti-CD28–stimulated T cells treated in the presence or absence of MP-04 (250 μM). Sequential injections of oligomycin, FCCP, and rotenone/antimycin A are indicated. **(B)** Quantification of glycolytic activity derived from ECAR measurements, demonstrating reduced glycolytic reliance in stimulated T cells treated with MP-04 compared with stimulated controls. **(C)** Real-time oxygen consumption rate (OCR) measured under the same experimental conditions. Each dot represents one donor sample. Data are presented as mean ± SEM. Statistical analysis was performed using one-way ANOVA with correction for multiple comparisons. *p < 0.05, **p < 0.01; ns, not significant.

To assess the effect of MP-04 on cellular metabolic parameters, extracellular acidification rate (ECAR) and oxygen consumption rate (OCR) were measured in human T cells under unstimulated and polyclonally stimulated conditions by anti-CD3 and anti-CD28 antibodies. Polyclonal activation of T cells resulted in an increase in ECAR compared with unstimulated cells, consistent with increased glycolytic activity under these conditions (Figures 3A,B). Treatment with MP-04 in stimulated T cells was associated with a reduction in ECAR relative to stimulated controls, while ECAR in unstimulated cells was not materially altered. Following sequential treatment with oligomycin and FCCP, ECAR increased in both stimulated and unstimulated cells, consistent with induction of maximal glycolytic capacity. The relative reduction in ECAR observed in stimulated cells treated with MP-04 was maintained across these conditions. In contrast, oxygen consumption rate (OCR) measurements did not demonstrate consistent or significant differences between stimulated and unstimulated cells, nor with MP-04 treatment under the conditions tested (Figure 3C).

### 3.5 MP-04 reduces cytokine production in polyclonally stimulated T cells *in vitro*

**FIGURE 4:**
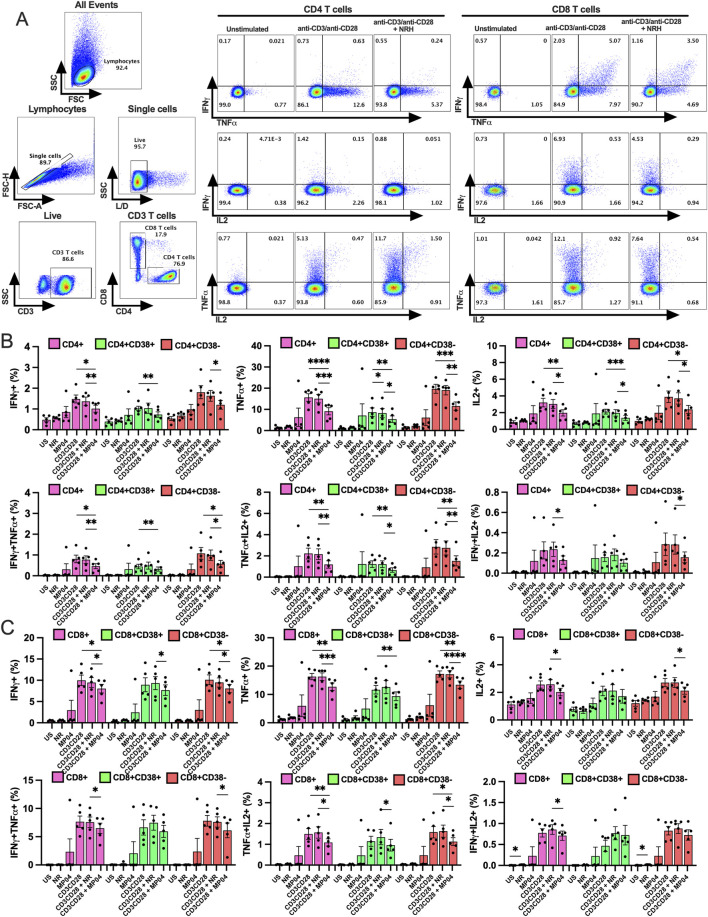
MP-04 more effectively reduces pro-inflammatory cytokine production than NR in activated T cells. **(A)** Representative gating strategy for identification of IFNγ^+^, TNFα^+^, and IL-2^+^ CD4^+^ and CD8^+^ T cells. **(B)** Direct comparison of frequencies of single- and dual-cytokine–producing CD4^+^ T cells following anti-CD3/anti-CD28 stimulation in the presence of MP-04 (250 μM) or NR (250 μM). MP-04 significantly reduced activation-induced TNFα^+^, IL-2^+^, and multifunctional CD4^+^ T cell responses relative to stimulated controls and demonstrated greater suppression compared with NR. **(C)** Direct comparison of frequencies of single- and dual-cytokine–producing CD8^+^ T cells under the same conditions. MP-04 markedly reduced IFNγ^+^, TNFα^+^, IL-2^+^, and IFNγ^+^/TNFα^+^ dual-positive CD8^+^ T cells, with a greater magnitude of effect than NR. Each dot represents one donor. Data are presented as mean ± SEM. Statistical analysis was conducted using repeated measures one-way ANOVA with the Geisser-Greenhouse correction and uncorrected Fisher’s LSD (*p < 0.05, **p < 0.01, ***p < 0.001; ns, not significant).

**FIGURE 5:**
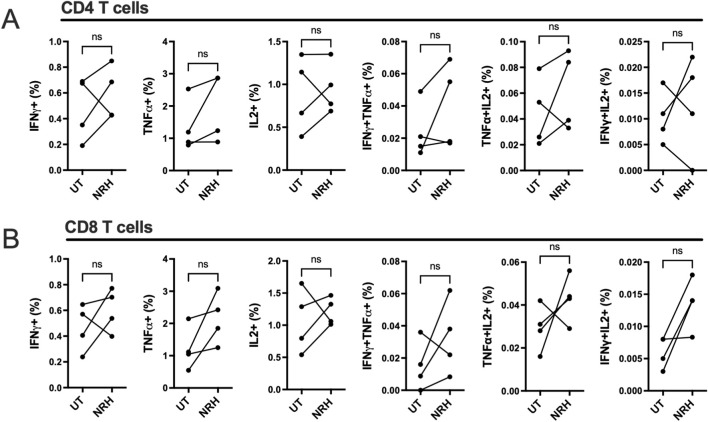
MP-04 does not alter basal cytokine production in unstimulated T cells. Percentages of cytokine-producing CD4^+^ **(A)** and CD8^+^ **(B)** T cells following MP-04 treatment (250 μM) in the absence of polyclonal stimulation. Each dot represents one donor. Statistical analysis was performed using paired t-test. Ns, not significant.

To evaluate the effect of MP-04 on T-cell activation, intracellular cytokine production was assessed in polyclonally stimulated CD4^+^ and CD8^+^ T cells. Polyclonal stimulation with anti-CD3 and anti-CD28 antibodies resulted in marked increases in intracellular cytokine production compared with unstimulated controls, including IFNγ (>3-fold in CD4^+^ and >10-fold in CD8^+^ T cells), TNFα (>10-fold in both subsets), and IL-2 (>2-fold in both subsets) (Figure 4). Treatment of stimulated T cells with MP-04 was associated with a reduction in cytokine-producing cells across multiple markers. In CD4^+^ T cells, MP-04 reduced the proportion of TNFα^+^ and IL-2^+^ cells, as well as multifunctional cytokine-producing populations, relative to stimulated controls. In CD8^+^ T cells, MP-04 treatment was associated with reductions in IFNγ^+^, TNFα^+^, IL-2^+^, and IFNγ^+^/TNFα^+^ dual-positive populations across donors (Figures 4B,C). Direct comparison under equivalent *in vitro* exposure conditions demonstrated that MP-04 produced a greater reduction in activation-induced cytokine production compared with NR at a similar concentration. Importantly, MP-04 did not materially alter basal cytokine expression in unstimulated T cells (Figure 5), indicating that the observed effects were specific to activated conditions.

### 3.6 MP-04 is associated with improved renal function and reduced inflammatory cytokine levels *in vivo*

**FIGURE 6:**
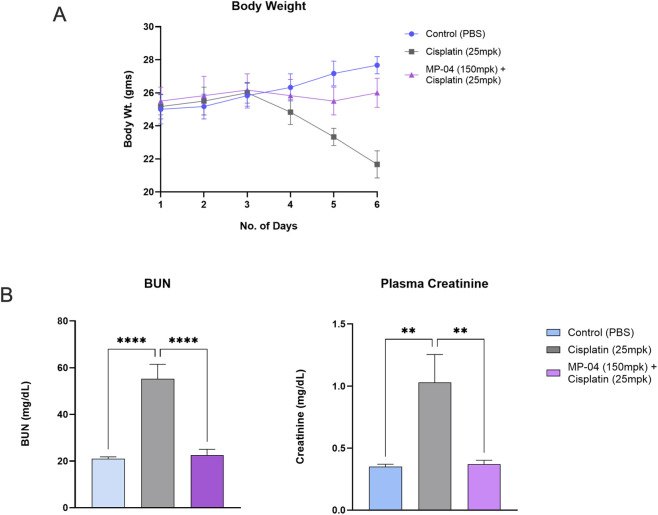
MP-04 preserves body weight and renal function in cisplatin-induced acute kidney injury. **(A)** Body weight change in mice receiving cisplatin with or without MP-04 treatment. **(B)** Serum creatinine and blood urea nitrogen (BUN) levels measured at study endpoint. Data are presented as mean ± SEM. Statistical analysis was performed using one-way ANOVA with multiple comparison correction. **p < 0.01, ****p < 0.0001.

**FIGURE 7:**
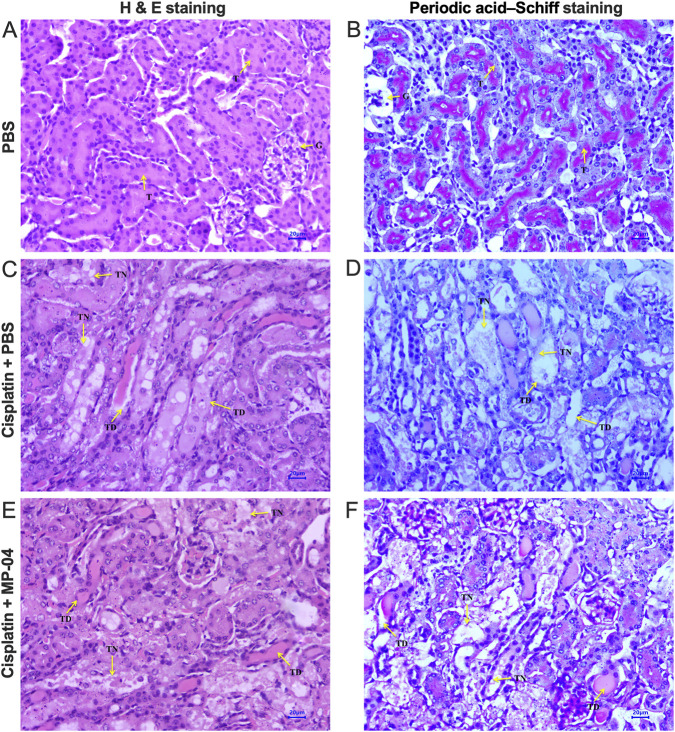
MP-04 attenuates cisplatin-induced renal histopathology. Representative kidney sections (400× magnification) stained with hematoxylin and eosin (H&E; **(A,C,E)**) and periodic acid–Schiff (PAS; **(B,D,F)**). **(A,B)** PBS control group showing normal renal cortex architecture. **(C,D)** Cisplatin + PBS group demonstrating tubular dilation, epithelial degeneration, necrosis, and loss of brush borders. **(E,F)** Cisplatin + MP-04 group showing reduced tubular injury compared with cisplatin alone. Arrows indicate renal tubules (T), glomeruli (G), tubular dilation (TD) and tubular degeneration and necrosis (TN).

**FIGURE 8:**
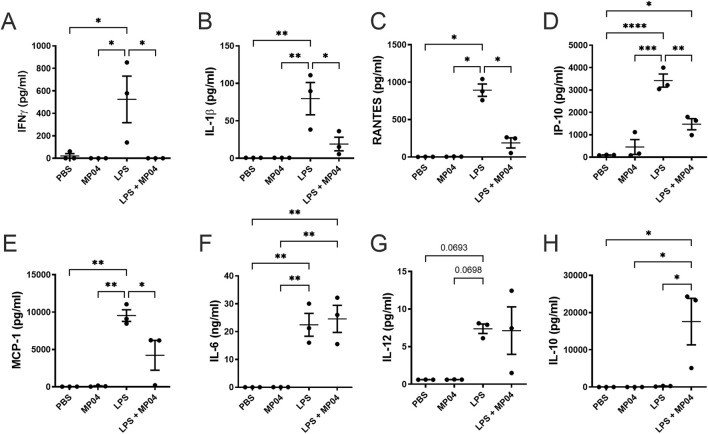
MP-04 attenuates systemic inflammatory cytokine responses in endotoxin-induced inflammation. Plasma concentrations of **(A)** IFNγ, **(B)** IL-1β, **(C)** RANTES, **(D)** IP-10, **(E)** MCP-1, **(F)** IL-6, **(G)** IL-12, and **(H)** IL-10 measured 6 h after treatment with PBS, MP-04, lipopolysaccharide (LPS), or MP-04 + LPS. Each dot represents 1 mouse. Data are shown as mean ± SEM. Statistical analysis was performed using one-way ANOVA with multiple comparison correction. *p < 0.05, **p < 0.01, ***p < 0.001.

The effect of MP-04 on renal injury was evaluated in a cisplatin-induced mouse model of acute kidney injury. Cisplatin-treated animals demonstrated progressive body weight loss and elevations in serum creatinine and blood urea nitrogen (BUN) at day 6 (Figure 6). Mice treated with MP-04 in the presence of cisplatin showed reduced body weight loss and lower serum creatinine and BUN levels compared with cisplatin-only controls. Histological analysis of kidney sections from cisplatin-treated animals demonstrated tubular dilation, epithelial degeneration, necrosis, and loss of brush borders. These changes were less pronounced in animals treated with MP-04, with preservation of tubular architecture relative to cisplatin-only controls (Figure 7). The effects of MP-04 on systemic inflammatory responses were evaluated in a lipopolysaccharide (LPS)-induced model. LPS administration resulted in increased circulating levels of multiple pro-inflammatory cytokines, including IFNγ, IL-1β, RANTES, IP-10, MCP-1, IL-6, and IL-12 at 6 h post-challenge (Figure 8). Pre-treatment with MP-04 was associated with reduced levels of several cytokines, including IFNγ, IL-1β, RANTES, IP-10, and MCP-1, compared with LPS-only controls. In contrast, IL-6 and IL-12 levels were not significantly altered under the conditions tested. An increase in IL-10 levels was also observed in MP-04-treated animals.

## 4 Discussion

Multiorgan failure associated with systemic inflammation is coupled with profound failure of cellular bioenergetics, characterized by the depletion of nicotinamide adenine dinucleotide (NAD^+^), a coenzyme essential for mitochondrial function, redox homeostasis, and genomic integrity (Brealey et al., 2002; Verdin, 2015; Hotchkiss et al., 2016; Jalan et al., 2020). In this context, strategies aimed at restoring NAD^+^ levels have emerged as a potential therapeutic approach. This study presents the preclinical evaluation of MP-04, an intravenous formulation of dihydronicotinamide riboside (NRH), with a focus on its ability to augment NAD^+^ levels, its associated biological effects *in vitro, ex vivo* and *in vivo* experimental systems, and extensive pharmacology and toxicology characterization to support clinical development.

A consistent finding across *in vitro*, *ex vivo*, and *in vivo* models was the magnitude and reproducibility of NAD^+^ elevation following MP-04 exposure. MP-04 produced greater increases in intracellular NAD^+^ levels compared with nicotinamide riboside under equivalent *in vitro* conditions, consistent with prior reports demonstrating the enhanced cellular uptake and metabolic conversion of NRH via adenosine kinase–dependent pathways (Yang and Sauve, 2016; Giroud-Gerbetant et al., 2019). *In vivo*, intravenous administration of MP-04 was associated with dose-dependent increases in NAD^+^ levels in circulation and tissues. Notably, NAD^+^ levels remained elevated beyond the period of detectable circulating MP-04, indicating a pharmacokinetic–pharmacodynamic profile characterized by transient systemic exposure providing sustained biological effect.

In addition to NAD^+^ augmentation, MP-04 treatment was associated with measurable biological effects in immune and organ injury models. In activated human T cells, MP-04 exposure was associated with reduced cytokine production across multiple markers. Similarly, in an endotoxin-induced inflammatory model, MP-04 treatment was associated with reduced circulating levels of several pro-inflammatory cytokines and increased levels of IL-10. In a cisplatin-induced acute kidney injury model, MP-04 treatment was associated with improvements in functional markers, including serum creatinine and blood urea nitrogen, as well as reduced histological evidence of tubular injury. These observations are consistent with prior reports linking NAD^+^ biology to inflammatory responses and organ injury (Angus and van der Poll, 2013; Deutschman and Tracey, 2014; Tran et al., 2016; Guan et al., 2017; Poyan Mehr et al., 2018).

While these findings demonstrate consistent associations between MP-04 exposure, NAD^+^ augmentation, and downstream biological effects, the current study does not establish a direct causal relationship between these observations. NAD^+^ is involved in multiple cellular processes, including regulation of redox state, mitochondrial metabolism, and activity of NAD^+^-dependent enzymes such as sirtuins and PARPs (Pearce et al., 2013; O'Neill et al., 2016; Buck et al., 2017). However, the present work did not include targeted molecular interrogation of these pathways, and therefore the relative contribution of specific mechanisms cannot be determined. Accordingly, the observed immunological and functional effects should be interpreted as associated with, but not definitively mediated by, NAD^+^ augmentation.

Similarly, extracellular flux analyses demonstrated changes in ECAR responses in activated T cells following MP-04 treatment. ECAR is a primary indicator of aerobic glycolysis in T cells, and its reduction generally correlates with decreased inflammatory activity (Pearce et al., 2013; O’Neill et al., 2016; Buck et al., 2017). However, these measurements do not provide direct evidence of intracellular metabolic flux or pathway-specific remodeling. As such, these findings indicate changes in metabolic parameters under the experimental conditions tested and strongly suggestive of immune modulation, additional experiments are needed to characterize specific alterations in oxidative phosphorylation or glycolytic pathways.

The IND-enabling program for MP-04 includes GLP-compliant toxicology studies in both rodent and dog, which demonstrated a favorable safety profile and large safety margins in the context of the anticipated clinical dose range. These data support progression to first-in-human studies. The initial clinical program will focus on evaluating safety, tolerability, pharmacokinetics, and pharmacodynamics in healthy volunteers. The proposed dose range of 10–500 mg is informed by preclinical toxicology, human equivalent dose projections, and prior clinical experience with related NAD^+^ precursors, including nicotinamide riboside and nicotinamide mononucleotide, which have demonstrated favorable safety profiles and dose-dependent increases in circulating NAD^+^ levels (Trammell et al., 2016; Martens et al., 2018; Conze et al., 2019; Irie et al., 2020; Yoshino et al., 2021). Measurement of circulating NAD^+^ and related metabolites will form a key component of pharmacodynamic assessment in these studies.

Limitations of the current study include the preclinical models which were limited to acute injury and pre-treatment paradigms, which do not address the effects of MP-04 in established disease. There is a limited characterization of molecular pathways and pharmacokinetic–pharmacodynamic characterization within disease models. Notwithstanding these limitations, the study demonstrates consistent and substantial NAD^+^ augmentation, accompanied by reproducible biological effects and a favorable safety profile in GLP-compliant toxicology studies in both rodent and non-rodent species, supporting the planned clinical development of IV administered MP-04.

The development of MP-04 as an intravenous NAD^+^ precursor is supported by its safety in GLP-toxicology studies and pharmacokinetic–pharmacodynamic profile, characterized by rapid systemic exposure associated with sustained elevation of NAD^+^ levels in circulation and tissues. These features provide a basis for further clinical evaluation, particularly in conditions where systemic inflammation and metabolic dysfunction contribute to organ injury. In this context, hepatorenal syndrome–associated acute kidney injury (HRS-AKI) represents a setting of high unmet need, where current therapies such as vasoconstrictors and albumin primarily target hemodynamic mechanisms and are associated with variable efficacy and limited tolerability (Jalan et al., 2012; Wong et al., 2021). The observed effects of MP-04 on NAD^+^ augmentation and associated biological responses in preclinical models support its investigation as a complementary approach in such conditions.

In conclusion, MP-04 is a potent intravenous NAD^+^ precursor that produces sustained increases in NAD^+^ levels and is associated with consistent biological effects across multiple preclinical systems. While the mechanistic basis of these effects remains to be further defined, the current data provides a foundation for clinical development and supports the evaluation of MP-04 in conditions characterized by systemic inflammation and metabolic stress, including HRS-AKI.

## Data Availability

The original contributions presented in the study are included in the article/supplementary material, further inquiries can be directed to the corresponding authors.
