# Supplementary figures and images for: Pharmacological profiling of intravenous MP-04: sustained NAD+ augmentation, immune modulation, and renal protection in preclinical models
Source: Front Pharmacol. 2026 Jun 10;17:1832979. doi: 10.3389/fphar.2026.1832979 (PMC13291046; doi:10.3389/fphar.2026.1832979)

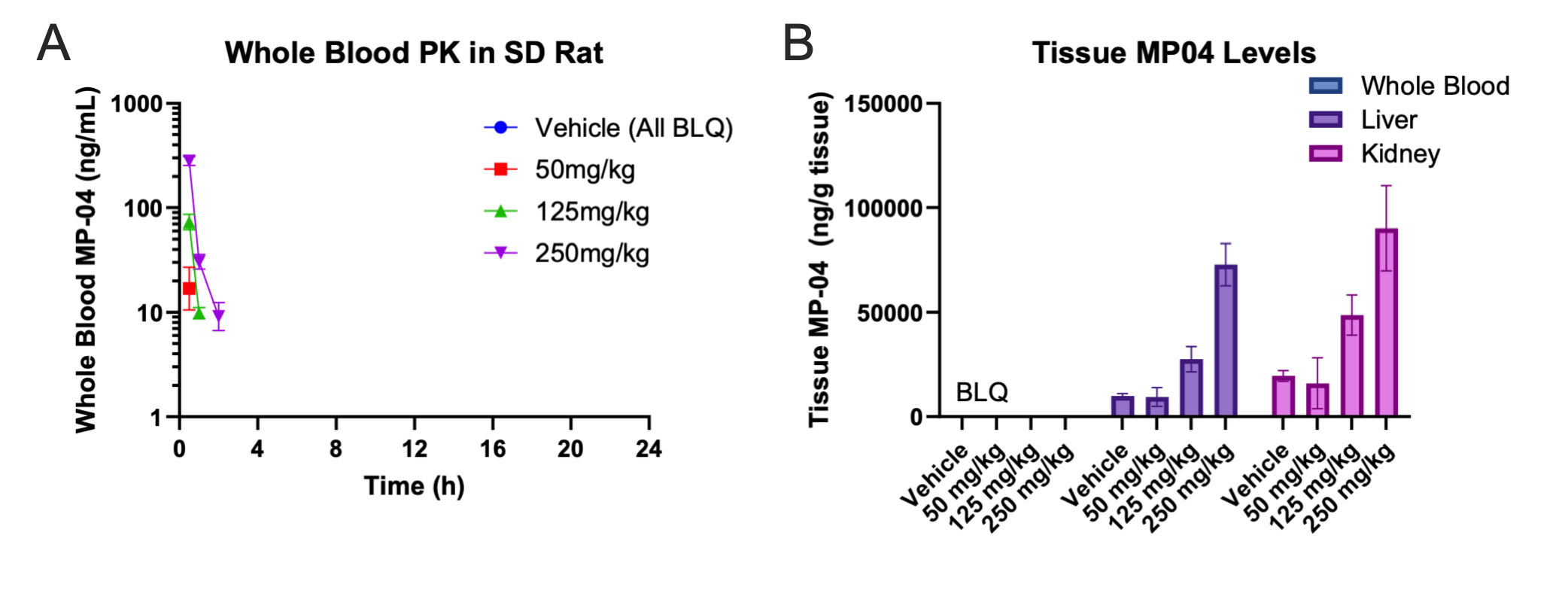

Supplement: Supplementary file 1 [file Image3.tiff]

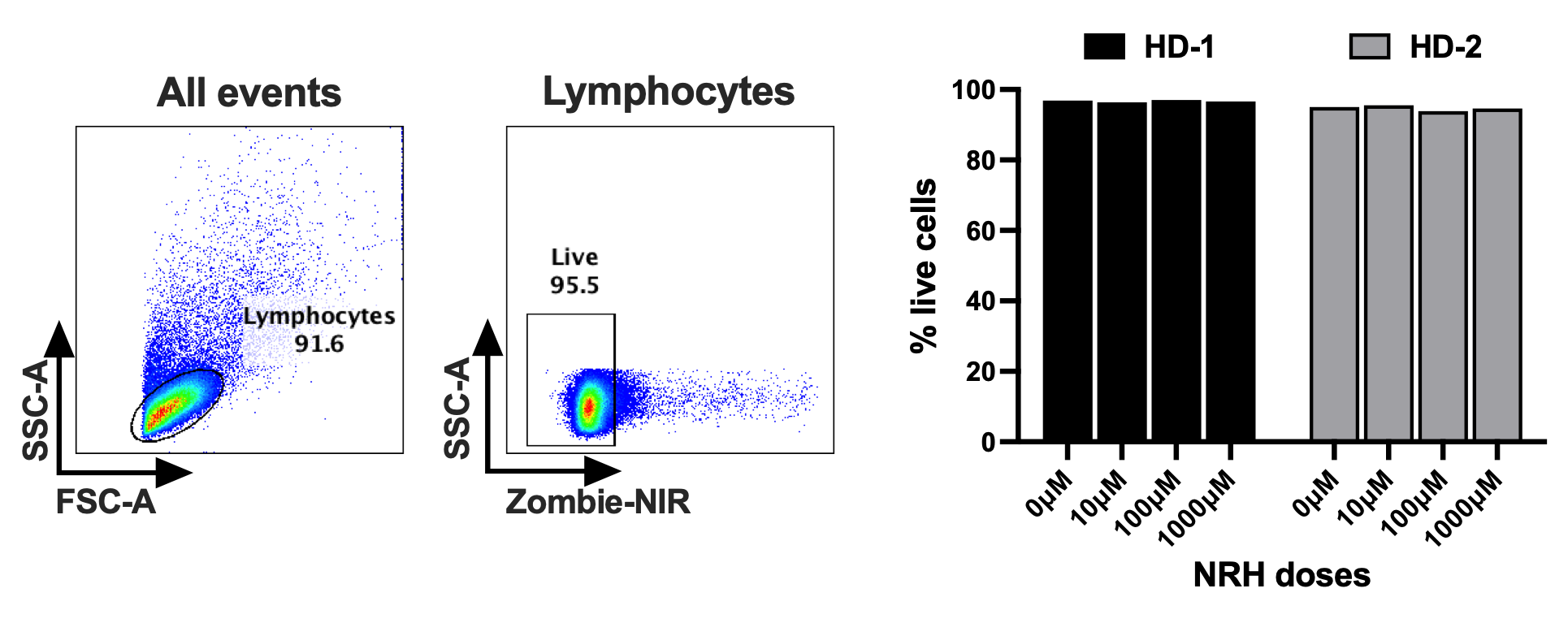

Supplement: Supplementary file 2 [file Image1.tiff]

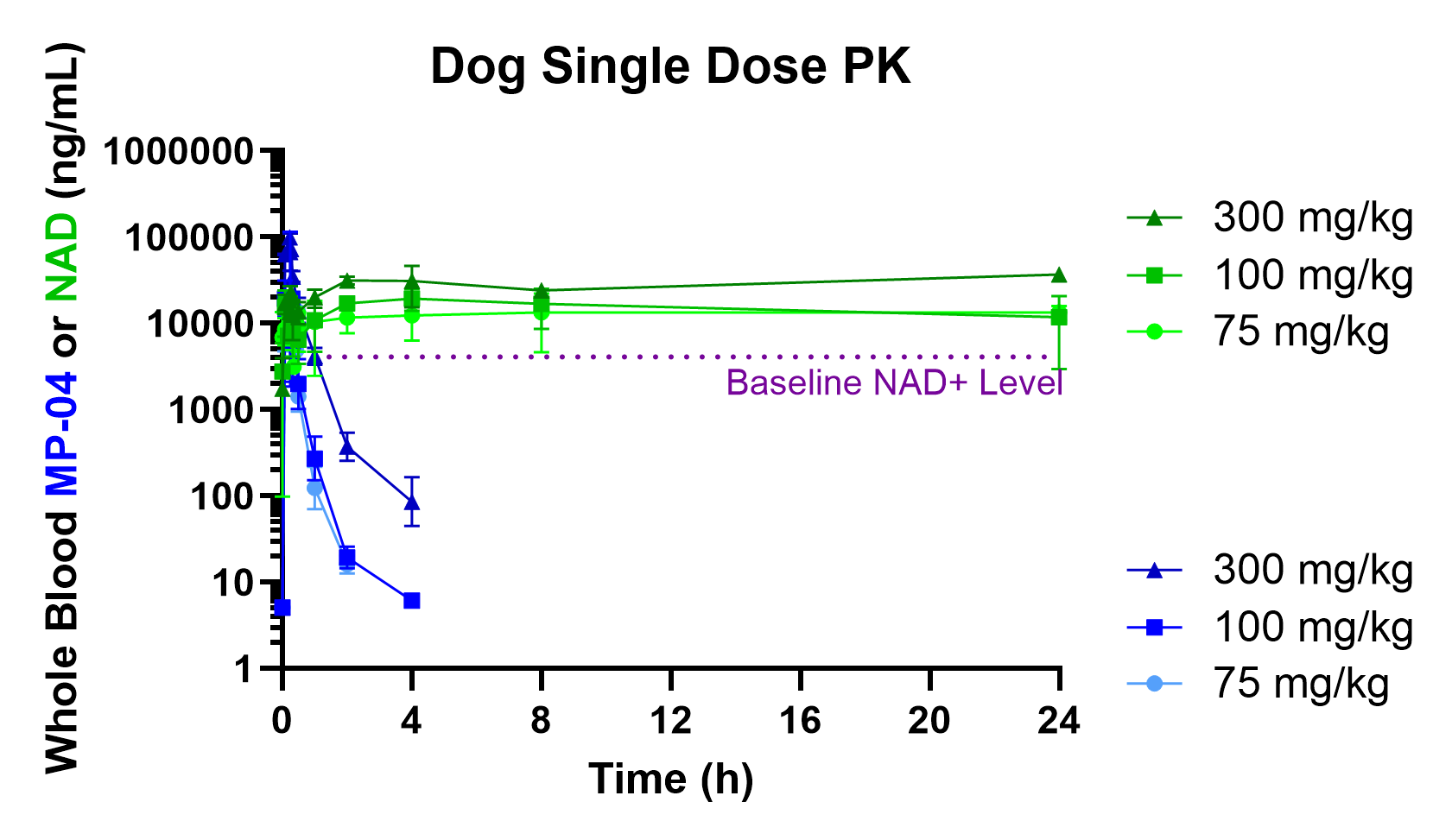

Supplement: Supplementary file 3 [file Image6.tif]

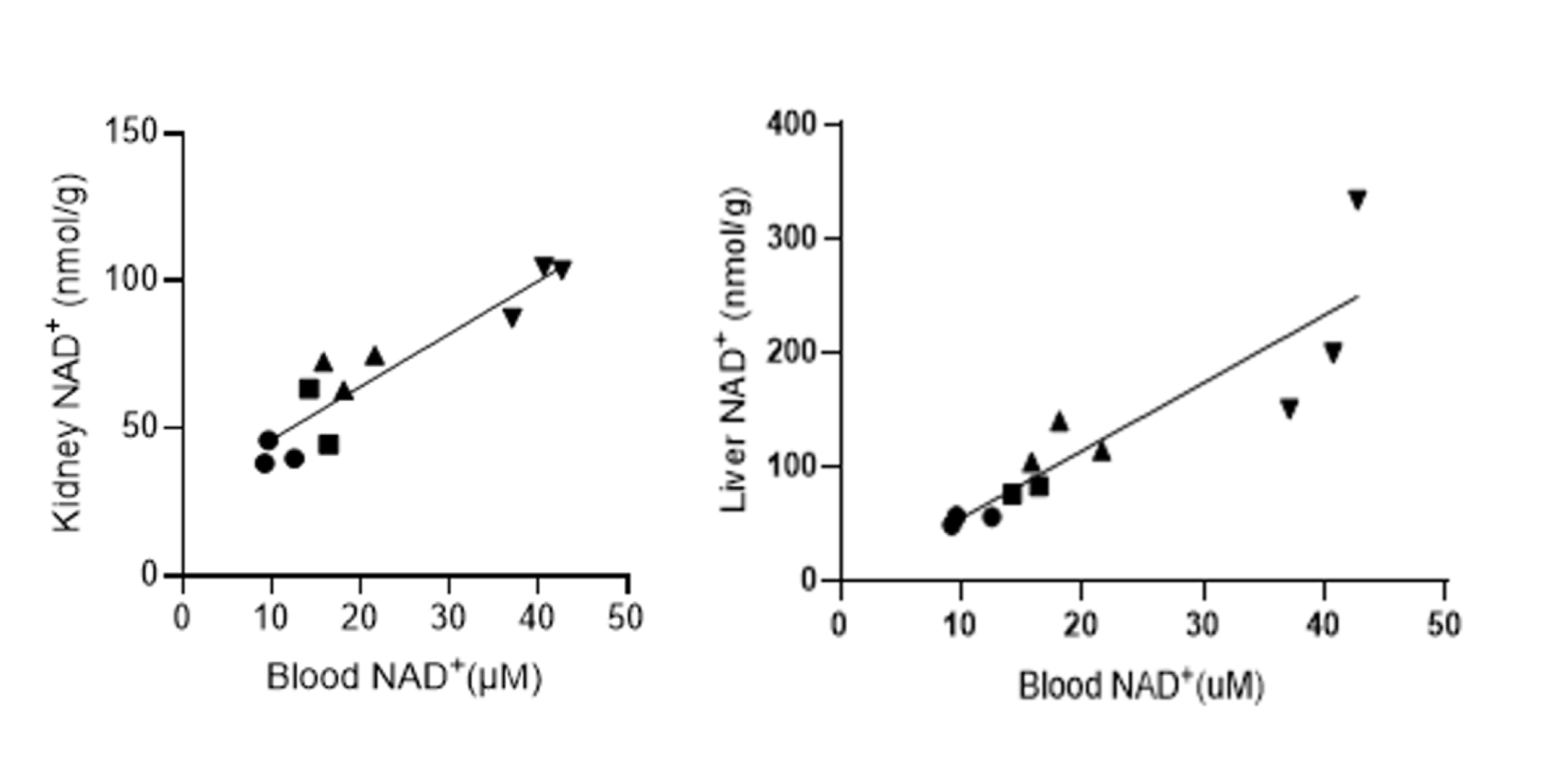

Supplement: Supplementary file 4 [file Image5.tiff]

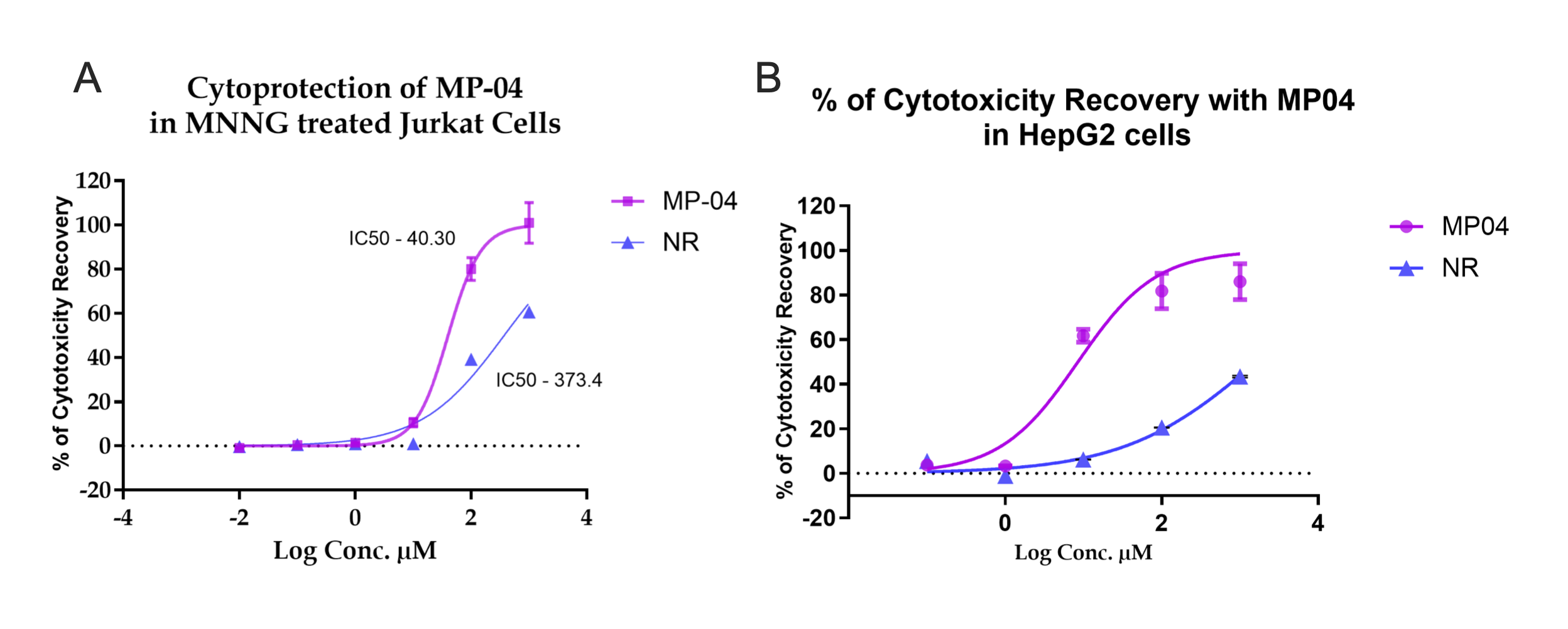

Supplement: Supplementary file 5 [file Image2.tiff]

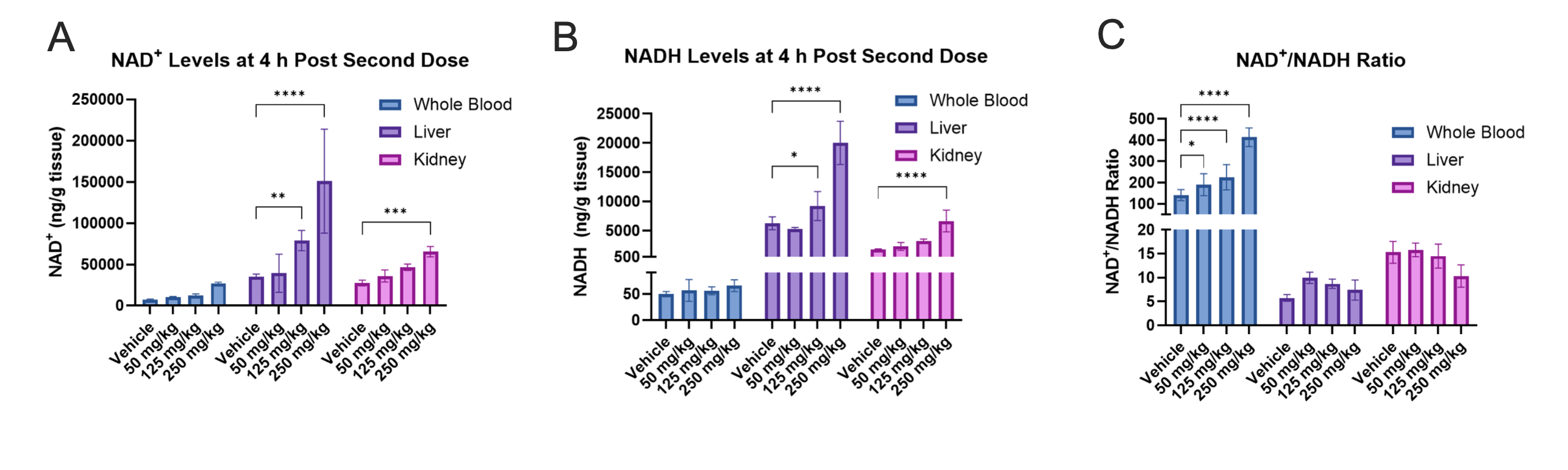

Supplement: Supplementary file 6 [file Image4.tiff]
